# Oak (*Quercus petraea*) Leaf-Based Kombucha: A Sustainable Approach to Fermented Beverages

**DOI:** 10.3390/foods15040707

**Published:** 2026-02-14

**Authors:** Tomas Pencak, Dani Dordevic, Dominika Kotianova, Fouad Ali Abdullah Abdullah, Galia Zamaratskaia

**Affiliations:** 1 Department of Plant Origin Food Sciences, Faculty of Veterinary Hygiene and Ecology, University of Veterinary Sciences Brno, Palackeho tr. 1946/1, 612 42 Brno, Czech Republic; h23448@vfu.cz (T.P.); dordevicd@vfu.cz (D.D.); kotianovad@vfu.cz (D.K.); 2 Department of Animal Origin Food & Gastronomic Sciences, Faculty of Veterinary Hygiene and Ecology, University of Veterinary Sciences Brno, Palackeho tr. 1946/1, 612 42 Brno, Czech Republic; abdullahf@vfu.cz; 3 Department of Molecular Sciences, Swedish University of Agricultural Sciences (SLU), 750 07 Uppsala, Sweden

**Keywords:** *Quercus*, fermentation, polyphenols, antioxidant capacity, functional beverage

## Abstract

Kombucha is traditionally produced by fermenting black tea (*Camellia sinensis*), but exploring alternative plant materials could enhance sustainability and resource diversity. The study evaluated the potential of oak (*Quercus petraea*) leaves as a substrate for kombucha fermentation. Fresh and processed oak leaves were used to prepare kombucha infusions fermented for different time periods and compared with black tea kombucha, as well as unfermented infusions. Fermentation increased total phenolic content and antioxidant capacity in the dried and crushed leaf treatments, while lowering pH, which confirms effective acidification. Overall, beverages produced from dried and crushed oak leaves exhibited phenolic and antioxidant characteristics comparable to those of black tea kombucha. These findings suggest that *Q. petraea* leaves may represent a sustainable (due to the vast amount of raw material), same as not traditional alternative for kombucha production, contributing to the diversification of fermentation substrates in functional beverage development.

## 1. Introduction

Fermentation is one of the oldest biotechnological processes and remains a key technique in modern food production, enhancing food safety, stability, and nutritional value [1,2]. Among fermented beverages, kombucha is produced by fermenting sweetened tea with a symbiotic culture of bacteria and yeast (SCOBY), which mainly contains acetic acid bacteria (*Komagataeibacter xylinus*, *Acetobacter pasteurianus*), lactic acid bacteria (*Lactobacillus* spp.), and yeasts (*Saccharomyces cerevisiae*, *Zygosaccharomyces bailii*). In recent years, kombucha has gained worldwide popularity for its functional properties, including antioxidant, antimicrobial, and potential health-promoting effects [3,4,5].

While black tea (*Camellia sinensis*) is traditionally used as the substrate, increasing interest in sustainable food systems has encouraged the search for alternative plant materials that could reduce reliance on conventional tea crops. Plant-derived substrates are among the most frequently used alternatives to traditional tea leaves. These include herbs, fruits, flowers, and various by-products, which are mainly chosen to provide unique sensory qualities and to enhance the beverage’s bioactive profile, thereby supporting its potential functional properties. Alternative substrates, such as rice and barley, have been explored for kombucha production. However, these studies have shown limitations, as the resulting beverages exhibited lower performance in biological parameters (yeast and acetic acid bacteria counts), chemical properties (acetic acid and ethanol content), and antioxidant activity compared to traditional black tea kombucha. Further studies focusing on the use of different types of tea (white, green, and yellow) reported higher total polyphenol content as well as greater antioxidant activity compared to black tea kombucha [6,7,8]. Kombuchas produced from various types of leaves, such as bamboo leaves, mulberry leaves, and coffee leaves, have demonstrated considerable potential for kombucha production, suggesting that leaves from different plants could also serve as suitable substrates [9,10].

Oak leaves (*Quercus* spp.) are rich in phenolic compounds, tannins, and other bioactive molecules with antioxidant and anti-inflammatory potential [11,12]. However, their application in kombucha production remains largely unexplored. Previous studies have investigated other oak species (e.g., *Q. resinosa*, *Q. robur*, *Q. pyrenaica*), but no data are available for *Q. petraea* (sessile oak), are widely distributed European species known for its unique phenolic profile [13].

In recent years, kombucha production has been increasing worldwide and it is now commercially available in a variety of flavours [14]. While traditional kombucha made from black tea has been extensively studied [15,16,17], some research suggests that different types of oak leaves could serve as an alternative to black tea in kombucha production [12,18]. *Q. petraea* is among the most widespread oak species in central Europe [19]. Utilization of local products is commonly associated with freshness, environmental sustainability, and support for local economies [20]. To the best of our knowledge, no study has been conducted on the production of kombucha using *Quercus petraea* leaves. For these reasons, *Q. petraea* leaves may represent a suitable alternative for kombucha production.

The aim of the study was to investigate the feasibility of using *Quercus petraea* leaves as an alternative substrate for kombucha production. The influence of leaf processing and fermentation time on key physicochemical and functional properties of the resulting beverages was evaluated in comparison with traditional black tea kombucha.

## 2. Materials and Methods

### 2.1. Starter Culture and Raw Materials

The kombucha starter culture (SCOBY) and 150 mL of starter liquid were obtained from a certified health food store Zdraví s chutí (Brno, Czech Republic). Commercial Ceylon black tea (*Camellia sinensis*, Grešík Valdemar, Brno, Czech Republic) was used as the control.

Fresh *Quercus petraea* leaves were collected in July 2024 from the Brno—Jundrov area, Czech Republic (49°12′48.0″ N, 16°32′47.9″ E). Collected leaves were washed, air-dried, and divided into three treatment groups: (a) fresh leaves (used immediately after washing), (b) dried leaves (dried at 40 °C for 24 h in a forced-air oven, Memmert UF110, Memmert, Schwabach, Germany), (c) dried and crushed leaves (dried as above and manually crushed to 1–10 mm particles). All samples were stored in airtight glass containers at room temperature until use.

### 2.2. Preparation of Infusions and Fermentation

Infusions were prepared by adding 10 g of sample (fresh, dried, or dried–crushed leaves, or black tea) to 1 L of boiling distilled water. The mixture was steeped for 10 min, filtered through Whatman No. 1 paper, and supplemented with 100 g sucrose (Cukrovar Vrbátky a.s., Vrbátky, Czech Republic). After cooling to 25 °C, each infusion was inoculated with 150 mL of kombucha starter and 15 g of SCOBY.

Fermentation was carried out in sterile 2 L glass jars covered with breathable paper towels, under aerobic conditions at 25 ± 1 °C and ambient light. Samples were collected after 7, 10, and 13 days of fermentation. Following sampling, fermentation was stopped by immediate freezing (–20 °C).

### 2.3. Preparation of Extracts for Spectrophotometric Analysis

The extraction procedure was performed to obtain phenolic and antioxidant compounds from both unfermented and fermented samples. Solid samples corresponded to unfermented oak leaves (fresh, dried, and dried–crushed), while liquid samples represented the produced kombucha beverages collected after 7, 10, and 13 days of fermentation.

For each analysis, 0.10 g of solid material or 0.10 mL of liquid sample was placed into a dark test tube, and 20 mL of a 50% (*v*/*v*) ethanol–water mixture was added. The mixture was extracted for 30 min in an ultrasonic water bath (Bandelin Sonorex, Berlin, Germany, 40 kHz, 25 °C) and then filtered through Whatman No. 1 paper (pore size 11 µm, to remove suspended particles before analysis, Maidstone, UK). The resulting extracts were immediately used for spectrophotometric analyses (DPPH, FRAP, and TPC). The methodology we used in our previous publication was employed for the preparation of extracts for spectrophotometric analysis [21].

### 2.4. Total Phenolic Content (TPC) Assessment

The TPC was measured using the Folin–Ciocalteu spectrophotometric method [22]. A 1 mL aliquot of extract was mixed with 5 mL of 10% (*v*/*v*) Folin–Ciocalteu reagent and 4 mL of 7.5% (*w*/*v*) Na_2_CO_3_. After 30 min incubation in the dark, absorbance was read at 765 nm using a UV–Vis spectrophotometer (Cecil Instruments, CE7210 DIET-QUEST, Cambridge, UK) with 10 mm quartz cuvettes. Calibration was performed with gallic acid (0–200 µg/mL, R^2^ = 0.999). Results were expressed as mg gallic acid equivalents (GAE) per g of sample.

### 2.5. DPPH Radical Scavenging Activity

Antioxidant activity was determined using the DPPH method. A 3 mL aliquot of extract was mixed with 1 mL of 0.1 mM DPPH solution (in ethanol) and incubated for 30 min in the dark. Absorbance was measured at 517 nm (Cecil Instruments, CE7210 DIET-QUEST, Cambridge, UK) with 10 mm quartz cuvettes. The blank consisted of 3 mL ethanol and 1 mL DPPH solution. The IC_50_ value, indicating the concentration needed to inhibit 50% of DPPH radicals, was calculated from the dose-response curve. Data are expressed as percentage inhibition and IC_50_ (mg/mL) [21].

### 2.6. Ferric Reducing Antioxidant Power (FRAP)

The FRAP reagent was prepared freshly by mixing 300 mM acetate buffer (pH 3.6), 10 mM TPTZ solution in 40 mM HCl, and 20 mM FeCl_3_·6H_2_O (10:1:1, *v*/*v*/*v*). An aliquot of 180 µL of extract was mixed with 3.9 mL of FRAP reagent and incubated at 37 °C for 8 min in the dark. Absorbance was measured at 593 nm (Cecil Instruments, CE7210 DIET-QUEST, Cambridge, UK) with 10 mm quartz cuvettes. A calibration curve was constructed using Trolox (6-hydroxy-2,5,7,8-tetramethylchroman-2-carboxylic acid) standards ranging from 0–1000 µM, with a correlation coefficient of R^2^ = 0.998. The results were expressed as µmol Trolox equivalents per g of sample [23].

### 2.7. Measurement of pH

The pH of kombucha and unfermented infusions was measured using a digital pH meter (Orion 4 STAR, Thermo Fisher Scientific, Waltham, MS, USA) calibrated at pH 4.00 and 7.00 before each use [21].

### 2.8. Colour Analysis

Colour characteristics including redness (a*), yellowness (b*), chroma (C* = √(a^2^ + b^2^)), and hue angle (h° = tan^−1^ (b*/a*)) were assessed for the kombucha samples. Measurements were carried out using a CM-5 spectrophotometer (Konica Minolta Sensing, Inc., Tokyo, Japan) following the CIE Lab* colour system. Data processing and parameter calculations were performed with SpectraMagic NX Color Data Software (version CM-S100w 2.03.0006, 2003–2010). Results are presented as the mean ± standard deviation (SD) from ten replicates per sample [24].

### 2.9. Statistical Analysis

All analyses were conducted in triplicate, and results were expressed as mean ± standard deviation (SD). Statistical differences were evaluated using one-way ANOVA in IBM SPSS Statistics, version 28.0 (IBM Corp., Armonk, NY, USA). Homogeneity of variances was verified using Levene’s test. Post-hoc comparisons were performed using Tukey’s test (equal variances) or Games–Howell test (unequal variances). Statistical significance was set at *p* < 0.05. Pearson correlations between selected parameters were also calculated using IBM SPSS Statistics.

## 3. Results and Discussion

The pH value is an important indicator of fermentation progress and product safety. In this study, all oak leaf kombucha samples exhibited a significant (*p* < 0.05) decrease in pH during fermentation (Table 1), confirming active microbial metabolism.

The pH of unfermented infusions ranged from 6.63 to 7.00, comparable to that of black tea (6.61). After fermentation, the pH decreased to values between 3.04 and 3.33, falling within the safe range for kombucha beverages (2.5–4.2) [25]. The fresh oak leaf kombucha showed the lowest final pH values (3.04–3.15), while the dried and crushed leaf kombucha exhibited slightly higher values (3.28–3.33). These statistically significant differences (*p* < 0.05) suggest that the pretreatment of leaves influences the rate and extent of acidification. Fresh leaves likely contained more readily fermentable compounds, resulting in faster microbial metabolism and acid production, whereas drying and crushing may have altered substrate composition, slowing acid release. These findings are consistent with previous reports that describe pH reduction as a reliable indicator of kombucha fermentation performance [26]. Hammel et al. [27] observed a similar rapid pH decline below 4.6 in black tea kombucha within 12 h, indicating early microbial activity sufficient to prevent the growth of foodborne pathogens. The antimicrobial effect of acidic kombucha environments against a broad range of bacteria has been well documented [28]. In comparison, Das et al. [29] and Xu et al. [30] reported higher pH values for unfermented tea infusions (5.29–6.4), confirming that fermentation substantially increases acidity. Estrada et al. [31] also demonstrated that starter inoculation immediately lowers pH to approximately 4.0 in oak leaf kombucha, similar to the initial conditions observed in our experiment. Although Vázquez-Cabral et al. [12] recorded even lower pH values (2.11–2.46) in kombucha obtained from *Q. resinosa* leaves. Overall, the pH evolution confirmed that *Q. petraea* leaves can support effective and safe kombucha fermentation. The observed variation between treatments likely reflects differences in the availability of fermentable substrates and buffering capacity among fresh and processed leaf materials.

Table 2 presents the total phenolic content of the plant material, infusions and kombucha samples after 7, 10 and 13 days of fermentation.

Phenolic compounds are secondary metabolites commonly found in many plant-based products, where, in addition to health benefits for human health, they also affect the aroma, taste, or colour of these products [32]. Due to their antioxidant properties, phenolics are considered one of the key bioactive compounds in kombucha, and fermentation has been shown to have a positive effect on their increase [33]. In our study, an increase (*p* < 0.05) in TPC content during fermentation was recorded in all three samples of oak leaf kombucha compared to unfermented infusions, with a statistically significant difference (*p* < 0.05) in two of them (dried and dried-crushed). In the control sample of tea kombucha, a decrease was recorded in all fermentation lengths. When observing total polyphenol content in relation to pH changes, it can be noted that in the oak leaf kombucha samples, which underwent more intensive fermentation (higher initial pH compared to the lower pH of the control), total polyphenol content increased, while in the less intensive fermentation of the control sample, a slight decrease was observed. This suggests that the more rapid acidification in the oak leaf infusions promoted the release or transformation of phenolic compounds. Overall, these results indicate a relationship between the rate of pH decrease and changes in polyphenol content during fermentation. Such an effect of pH has been demonstrated during wine fermentation, where it was observed that lower pH enhanced the extraction of certain phenolic compounds, such as anthocyanins and tannins [34]. The lowest TPC values were found in kombucha made from fresh oak leaves; however, pretreatments such as drying and crushing before infusion preparation enhanced the phenolic content. Notably, the kombucha prepared from dried and crushed leaves reached similar values to those of the black tea kombucha. Black tea manufacture usually involves processes such as rolling, fermentation and drying. Drying is a significant step that reduces water content, prolongs shelf life and promotes aroma formation [35]. Though, opposite findings and statements were observed by other authors: the content of phenolics is higher in fresh parts of plants due to their degradation during drying. Otherwise, it was also stated that the dried parts contain a higher content of antioxidants and phenolic substances compared to fresh samples [36,37,38]. The total polyphenol content of oak leaf kombucha (*Q. pyrenaica* and *Q. robur*) was also observed in a study by Estrada et al. [31], where the authors reported that kombucha made from oak leaves contained lower TPC values than those of the black tea kombucha control. The greater differences between the oak leaf kombucha and the control could be due to the use of a different oak species (*Q. petraea* in our study) or to the type of tea used for the control, as the same amount of raw material was used for kombucha production in our study. Contrary to our results, fermentation had no effect on TPC in oak leaf kombucha, which was unchanged during fermentation (0 to 14 days).

The FRAP assay results (Table 3) showed that the highest reducing power was observed in the black tea kombucha samples. Among the oak leaf treatments, a statistically significant increase (*p* < 0.05) in FRAP values was recorded for dried and dried–crushed leaves, indicating that pretreatment strongly influences antioxidant potential. However, this effect may result from water loss during drying, which concentrates the antioxidant compounds. In contrast, fresh oak leaf kombucha exhibited no measurable FRAP activity, suggesting limited release of redox-active compounds from intact tissue. This may be due to the lower solubility of phenolic compounds in water from fresh leaves or the insufficient breakdown of cell structures, which is consistent with previous studies showing that drying and mechanical processing enhance the extraction of plant materials [39]. The FRAP value for the dried crushed oak leaf kombucha increased from 0.10 to 0.23 µmol Trolox/g after fermentation, highlighting the beneficial effect of both processing and microbial transformation. These findings suggest that mechanical disruption and drying promote the liberation of phenolic antioxidants and facilitate microbial enzyme access to bound polyphenols. Similar fermentation-related improvements in antioxidant capacity were observed in kombucha prepared from black, green, and Pu’er teas [40], whereas Saimaiti et al. [41] reported no significant change in FRAP activity in vine and sweet tea kombucha, supporting the idea that substrate composition critically affects antioxidant outcomes.

Antioxidant capacity is an important indicator of the functional potential of fermented beverages and can be assessed through several complementary assays, including DPPH and FRAP. Since these methods rely on different reaction mechanisms, radical scavenging in DPPH and ferric ion reduction in FRAP, they may produce variable results depending on the type and reactivity of antioxidants present in the sample [33,42,43]. This could also explain why no antioxidant activity was detected by FRAP in the fresh leaf sample, whereas DPPH revealed measurable activity from day 10 of fermentation. Therefore, interpreting the overall trend across methods provides a more accurate evaluation of antioxidant behaviour during fermentation. Fermentation can enhance antioxidant properties through multiple mechanisms, such as the release of phenolic compounds from plant matrices, the synthesis of microbial metabolites with antioxidant potential, and the enzymatic conversion of phytochemicals into more active forms [44]. This was also reflected in our study, where the samples with the highest total phenolic content (dried and crushed oak leaf sample and black tea control) exhibited the highest antioxidant activity across both methods.

The DPPH assay results revealed significantly lower radical scavenging activity in all oak leaf kombucha samples compared with the black tea control (*p* < 0.05). Nevertheless, the same trend of enhancement with fermentation and pretreatment was evident. The dried and crushed oak leaf kombucha demonstrated the highest activity, increasing from 15.85% to 28.71% inhibition between days 7 and 13, whereas fresh leaf kombucha achieved only minimal inhibition (5.27% on day 7). These differences confirm that phenolic extractability and microbial accessibility play important roles in determining antioxidant outcomes. Although the DPPH values of oak leaf kombucha were lower than those reported in the literature for other substrates, such as black tea (61.4–78.6%) [45], purple basil (64.19–68.09%) [46], and stevia leaves (65.43–73.31%) [47], the consistent increase observed across fermentation days might indicate a similar pattern of bioactive compound release. The gradual enhancement in antioxidant capacity during fermentation likely results from microbial degradation of high-molecular-weight tannins into smaller, more active phenolic acids and flavonoids, as well as the formation of new antioxidant metabolites. This bioconversion process explains the positive correlation between fermentation time and both FRAP and DPPH results in the dried and crushed oak leaf samples.

Color is one of the most important visual attributes of beverages and represents a key factor in their overall quality [18]. Changes in the monitored colour parameters during fermentation are presented in Table 4, showing statistically significant differences (*p* < 0.05). These colour changes reflect polyphenol oxidation and pigment polymerization processes that are typical of kombucha fermentation [48]. The highest a* and b* values were recorded for the control sample (black tea), which maintained intense red and yellow tones throughout the fermentation process. In the kombucha samples prepared from oak leaves, a similar trend was observed for both parameters when comparing fresh, dried, and dried crushed leaves: a* and b* increased slightly, with noticeable differences mainly in yellowness, while the differences in redness were smaller, although all values remained below those of the black tea. The highest C* values were recorded for the control sample, which maintained an intense colour throughout the fermentation process. In the kombucha samples from oak leaves, C* increased gradually from the fresh to the more processed leaves. This trend parallels the a* and b* values, with colour saturation increasing alongside higher red and yellow components, although even the most saturated kombucha sample did not reach the intensity of the control sample. The hue angle (h°) results indicate that the colour change from infusion to fermented kombucha was much more pronounced in samples prepared from oak leaves than in those from black tea. In the oak leaf samples, the hue shifted noticeably during fermentation, reflecting significant changes in the beverage’s visual appearance. In contrast, the black tea showed little to no change, indicating that its colour remained largely stable. This demonstrates that the type and processing of the plant material strongly influence the extent of colour transformation during kombucha fermentation.

Pearson correlations of selected parameters (pH, total phenolic content, and color parameters) are shown in Table 5. Although not statistically significant, a strong negative trend was observed between pH and TPC in oak leaf kombucha samples. In contrast, the control showed a positive trend, suggesting a contrasting behavior of polyphenol dynamics across fermentation times, compared to the control. Statistically significant correlations (*p* < 0.05) were observed in nearly all samples when comparing fermentation trends of pH with color parameters. The strongest correlations were observed between pH and the red–green coordinate (a*) and hue angle (h°), with statistically significant correlations found in all three oak leaf kombucha samples. A positive correlation between pH and a* indicates that as pH decreased during fermentation, the a* also decreased, showing a shift towards green. On the other hand, the negative correlation with hue angle (h°) shows that decreasing pH corresponded to higher hue values, confirming that changes in pH during fermentation influenced the overall color tone of the kombucha beverage. The decrease in pH during fermentation may alter the color of kombucha, as well as the biological activity of the microbial consortium, which may also modify or even break down tea-derived pigments, meaning that much of the kombucha’s color comes from polyphenol derivatives [48]. These results indicate that kombucha color is influenced by multiple factors, reflecting the overall dynamics of the fermentation process.

## 4. Conclusions

The study demonstrated, for the first time, that oak leaves (*Quercus petraea*) can serve as a viable and sustainable substrate for kombucha production, offering an innovative alternative to traditional tea leaves. Fermentation of *Q. petraea* leaves, particularly when dried and crushed before infusion preparation, led to a significant increase in total phenolic content and antioxidant capacity, with values approaching those of conventional black tea kombucha. In contrast, beverages prepared from fresh leaves exhibited lower phenolic and antioxidant levels, confirming the importance of substrate pretreatment in optimizing bioactive compound release. The consistent acidification across all oak leaf variants indicates that the fermentation process was both microbiologically safe and effective. Although the absolute antioxidant values were lower than those of black tea, the observed positive trends highlighted the strong potential of *Q. petraea* as a functional raw material for developing regionally sourced, sustainable, and phenolic rich kombucha beverages. Future studies should include deeper analytical work, such as sensory profiling and consumer acceptance testing, to better characterize the phytochemical composition, aroma attributes, and consumer response to oak-leaf kombucha. Shorter fermentation trials may also help optimize flavor balance while maintaining desirable functional properties.

## Figures and Tables

**Table 1:** pH values of kombucha, infusions and plant material.

pH (*n* = 6)				
Plant Material	Infusions	7 Days	10 Days	13 Days
Fresh oak leaf	7.00 ± 0.05 ^aA^	3.04 ± 0.02 ^aB^	3.15 ± 0.04 ^aC^	3.13 ± 0.01 ^aC^
Dried oak leaf	6.68 ± 0.03 ^bA^	3.27 ± 0.06 ^bB^	3.24 ± 0.01 ^bB^	3.19 ± 0.01 ^bB^
Dried and crushed oak leaf	6.63 ± 0.04 ^bA^	3.33 ± 0.01 ^bcB^	3.31 ± 0.02 ^cB^	3.28 ± 0.02 ^cB^
Black tea	6.61 ± 0.02 ^bA^	3.37 ± 0.03 ^cB^	3.31 ± 0.02 ^cC^	3.22 ± 0.01 ^bD^

Different lowercase letters in the superscript indicate a statistically significant difference (*p* < 0.05) between samples for each column. Different uppercase letters in the superscript indicate a statistically significant difference (*p* < 0.05) between the samples for each row.

**Table 2:** Total phenolic content of kombucha, infusions and plant material.

Total Phenolic Content (mg GAE/g) (*n* = 6)					
Plant Material	Solid Samples	Infusions	7 Days	10 Days	13 Days
Fresh oak leaf	6.30 ± 0.10 ^aA^	0.05 ± 0.06 ^aBC^	0.08 ± 0.01 ^aB^	0.11 ± 0.01 ^aC^	0.10 ± 0.01 ^aC^
Dried oak leaf	16.66 ± 2.02 ^bA^	0.07 ± 0.03 ^aB^	0.12 ± 0.00 ^bC^	0.14 ± 0.00 ^bD^	0.16 ± 0.02 ^bD^
Dried and crushed oak leaf	27.55 ± 0.95 ^cA^	0.07 ± 0.00 ^aB^	0.15 ± 0.00 ^cC^	0.21 ± 0.05 ^bCD^	0.21 ± 0.01 ^cD^
Black tea	55.83 ± 3.75 ^dA^	0.27 ± 0.01 ^bB^	0.25 ± 0.03 ^dBC^	0.20 ± 0.08 ^abBC^	0.23 ± 0.01 ^cC^

Different lowercase letters in the superscript indicate a statistically significant difference (*p* < 0.05) between samples for each column. Different uppercase letters in the superscript indicate a statistically significant difference (*p* < 0.05) between the samples for each row.

**Table 3:** Antioxidant capacity of kombucha, infusions, and plant material.

**FRAP (µmol/g Trolox) (*n* = 6)**					
**Plant Material**	**Solid Samples**	**Infusions**	**7 Days**	**10 Days**	**13 Days**
Fresh oak leaf	122.43 ± 3.48 ^a^	n.d.	n.d.	n.d.	n.d.
Dried oak leaf	332.82 ± 8.78 ^bA^	n.d.	n.d.	0.17 ± 0.03 ^aB^	n.d.
Dried and crushed oak leaf	363.36 ± 3.86 ^cA^	n.d.	0.10 ± 0.03 ^aB^	0.23 ± 0.04 ^bC^	0.14 ± 0.03 ^aB^
Black tea	468.49 ± 9.90 ^dA^	n.d.	2.00 ± 0.10 ^bB^	1.62 ± 0.02 ^cC^	3.32 ± 0.04 ^bD^
**DPPH (% of Radical Inhibition) (*n* = 6)**					
**Plant Material**	**Solid Samples**	**Infusions**	**7 Days**	**10 Days**	**13 Days**
Fresh oak leaf	90.28 ± 0.11 ^aA^	n.d.	n.d.	5.27 ± 0.07 ^aB^	0.56 ± 0.33 ^aC^
Dried oak leaf	82.10 ± 0.04 ^bA^	n.d.	3.79 ± 1.73 ^aB^	10.90 ± 3.50 ^bC^	6.52 ± 0.44 ^bC^
Dried and crushed oak leaf	67.22 ± 0.15 ^cA^	35.70 ± 1.90 ^aB^	15.85 ± 2.45 ^bC^	28.71 ± 0.19 ^cD^	23.84 ± 0.11 ^cE^
Black tea	82.06 * ± 0.18	47.98 ± 10.90 ^bABC^	57.65 ± 1.43 ^cA^	54.81 ± 0.94 ^dB^	62.15 ± 2.81 ^dC^

Different lowercase letters in the superscript indicate a statistically significant difference (*p* < 0.05) between samples for each column. Different uppercase letters in the superscript indicate a statistically significant difference (*p* < 0.05) between the samples for each row. The sample marked with * was diluted 10 times and was not included in the statistical analysis. n.d.—not detected.

**Table 4:** Colour parameters (a*, b*, C*, h°) of Kombucha samples.

Plant Material	Infusion	7 Days	10 Days	13 Days
	a* (*n* = 10)			
Fresh oak leaf	1.54 ± 0.38 ^dA^	−0.78 ± 0.13 ^cB^	−0.73 ± 0.08 ^cB^	−0.80 ± 0.11 ^bB^
Dried oak leaf	2.72 ± 0.38 ^bA^	−0.97 ± 0.10 ^bC^	−0.87 ± 0.12 ^bBC^	−0.76 ± 0.11 ^bB^
Dried and crushed oak leaf	2.25 ± 0.21 ^cA^	−0.68 ± 0.14 ^cB^	−0.53 ± 0.15 ^dB^	−0.29 ± 0.15 ^cC^
Black tea	3.67 ± 0.13 ^aA^	5.46 ± 0.32 ^aB^	3.51 ± 0.21 ^aA^	4.32 ± 0.41 ^aC^
	b* (*n* = 10)			
Fresh oak leaf	4.79 ± 0.99 ^bA^	1.77 ± 0.75 ^dB^	1.19 ± 0.46 ^dB^	1.74 ± 0.61 ^dB^
Dried oak leaf	3.77 ± 0.34 ^cA^	3.43 ± 0.65 ^cA^	3.93 ± 1.24 ^cAB^	4.73 ± 0.60 ^cB^
Dried and crushed oak leaf	2.92 ± 0.20 ^dA^	6.91 ± 0.35 ^bB^	7.38 ± 0.43 ^bB^	8.31 ± 0.32 ^bC^
Black tea	8.93 ± 0.12 ^aA^	12.25 ± 0.89 ^aB^	9.70 ± 0.40 ^aC^	12.14 ± 1.26 ^aB^
	C* (*n* = 10)			
Fresh oak leaf	5.03 ± 1.05 ^bA^	1.95 ± 0.72 ^dB^	1.43 ± 0.36 ^dB^	1.95 ± 0.47 ^dB^
Dried oak leaf	4.65 ± 0.49 ^bA^	3.58 ± 0.60 ^cB^	4.04 ± 1.18 ^cAB^	4.80 ± 0.57 ^cA^
Dried and crushed oak leaf	3.69 ± 0.26 ^cA^	6.95 ± 0.33 ^bB^	7.40 ± 0.42 ^bC^	8.31 ± 0.31 ^bD^
Black tea	9.66 ± 0.12 ^aA^	13.41 ± 0.95 ^aB^	10.32 ± 0.44 ^aC^	12.89 ± 1.33 ^aB^
	h° (*n* = 10)			
Fresh oak leaf	72.28 ± 1.20 ^aA^	116.00 ± 7.43 ^aB^	124.63 ± 14.49 ^aB^	117.70 ± 14.43 ^aB^
Dried oak leaf	54.34 ± 1.60 ^cA^	106.45 ± 4.73 ^bB^	104.16 ± 6.68 ^bBC^	99.36 ± 2.36 ^bC^
Dried and crushed oak leaf	52.51 ± 2.01 ^dA^	95.67 ± 1.36 ^cB^	94.14 ± 1.33 ^cB^	92.00 ± 1.05 ^cC^
Black tea	67.64 ± 0.72 ^bA^	65.97 ± 0.40 ^dB^	70.13 ± 0.54 ^dC^	70.39 ± 0.33 ^dC^

Different lowercase letters in the superscript indicate a statistically significant difference (*p* < 0.05) between samples for each column. Different uppercase letters in the superscript indicate a statistically significant difference (*p* < 0.05) between the samples for each row.

**Table 5:** Pearson correlations (r) of selected parameters (pH, TPC, and color) of kombucha beverage samples across fermentation times.

Relation	Fresh Oak Leaf	Dried Oak Leaf	Dried and Crushed Oak Leaf	Black Tea
pH/TPC	−0.870	−0.914	−0.909	0.733
pH/a*	1.000 *	0.997 *	0.992 *	−0.413
pH/b*	0.984 *	−0.269	−0.973 *	−0.720
pH/C*	0.986 *	0.421	−0.963 *	−0.684
pH/h°	−0.984 *	−0.988 *	−0.996 *	−0.311
TPC/a*	−0.875	−0.885	−0.859	0.296
TPC/b*	−0.931	0.621	0.956 *	−0.076
TPC/C*	−0.925	−0.036	0.958 *	−0.013
TPC/h°	0.935	0.850	0.877	−0.713

r—Pearson correlation coefficient; * significant correlation (*p* < 0.05).

## Data Availability

The original contributions presented in this study are included in the article. Further inquiries can be directed to the corresponding author.
